# A ceRNA network-mediated over-expression of cuproptosis-related gene SLC31A1 correlates with poor prognosis and positive immune infiltration in breast cancer

**DOI:** 10.3389/fmed.2023.1194046

**Published:** 2023-05-18

**Authors:** Weibin Lian, Peidong Yang, Liangqiang Li, Debo Chen, Chuan Wang

**Affiliations:** ^1^Department of Breast Surgery, Quanzhou First Hospital Affiliated to Fujian Medical University, Quanzhou, Fujian, China; ^2^Department of Breast Surgery, Fujian Medical University Union Hospital, Fuzhou, Fujian, China

**Keywords:** SLC31A1, breast cancer, ceRNA, prognosis, immune infiltrates, m6A

## Abstract

**Introduction:**

Solute carrier family 31 member 1(SLC31A1) has been reported as the copper importer, and was identified to be involved in the process of “cuproptosis”. However, the mechanism of SLC31A1 in breast cancer remains unclear.

**Methods:**

We examined the expression of SLC31A1 mRNA in breast cancer tissues and cell lines using Real-time PCR. The data for this study were obtained from The Cancer Genome Atlas (TCGA) database and analyzed via R 3.6.3. TIMER, UALCAN, GEPIA2, STRING, Metascape, Kaplan–Meier Plotter, starBase and miRNet websites were used for a comprehensive analysis of SLC31A1.

**Results:**

Our study suggested that SLC31A1 mRNA was over-expressed in breast tumor tissue and breast cancer cell lines, and which was closely related to poor relapse-free survival (RFS) and distant metastasis-free survival (DMFS). In addition, we constructed a co-expression network of SLC31A1. Functional enrichment analysis indicated that they were mainly involved in copper ion transport. Interestingly, SLC31A1 expression was positively associated with all m6A-related genes, especially with YTHDF3 (*r* = 0.479). Importantly, the LINC00511/miR-29c-3p/SLC31A1 axis was identified as the most potential pathway promoting breast cancer progress by affecting copper transport. Furthermore, the expression level of SLC31A1 in breast cancer was positively correlated with tumor immune cell infiltration, immune cell biomarkers and cancer-associated fibroblast (CAF).

**Conclusion:**

Up-regulation of SLC31A1 expression and regulation of copper ion transport mediated by LINC00511-miR-29-3p axis is related to poor prognosis and positively correlated with tumor immune infiltration in breast cancer.

## Background

As the Global Cancer Statistics 2020 reported, breast cancer (BRCA) had become the world’s most commonly diagnosed cancer (1). Although there are many comprehensive treatments including surgery, chemotherapy, radiotherapy, and targeted therapy, some people still experience recurrence and death. The development of novel biomarkers and more effective treatments for breast cancer is needed desperately.

Recently, the term “cuproptosis” was first proposed by Peter et al. and is considerably different from other cell death types (2). Solute carrier family 31 member 1 (SLC31A1) has been reported as the copper importer and was identified as a critical gene involved in the process of “cuproptosis” (2, 3). SLC31A1 over-expression induces a distinct form of necrotic cell death in cells, i.e., cuproptosis, which differed from the classical cell death entities known. Caroline et al. have reported that the genetic loss of SLC31A1 decreased the viability and clonogenic survival of human hepatocellular carcinoma cell lines (4). The study of Yu et al. identified SLC31A1 as a regulatory node mediating copper ion transport and revealed a mechanism through which copper promotes pancreatic cancer progression (5). However, as a copper death-related gene, the mechanism of SLC31A1 in breast cancer remains unclear.

Numerous studies have shown that long non-coding RNA (lncRNA) or microRNA (miRNA) promotes breast cancer progression by regulating target genes (6, 7). Competing endogenous RNA (ceRNA) is an acting element capable of competitive binding to RNA. Previous studies indicated that ceRNA networks could link the functions of protein-coding mRNAs with non-coding RNA (ncRNA) functions to promote tumorigenesis (8). In this study, we predicted the regulation of non-coding RNA (ncRNA) related to SLC31A1 in breast cancer and attempted to construct a ceRNA network. In addition, a recent study from Chen et al. showed that mRNA N6-methyladenosine (m6A) could promote lung metastasis of breast cancer by affecting the translation efficiency of KRT7 (9). Therefore, we try to explore the correlation between the SLC31A1 expression and m6A-related genes. At present, programmed death receptor 1 (PD-1) and programmed death ligand 1 (PD-L1) immune checkpoint inhibitors have been approved for the treatment of triple-negative breast cancer. Our study further explores the connection between SLC31A1 and tumor immune infiltration to search for more suitable immunotherapy targets.

## Materials and methods

### Expression and survival analysis

Solute carrier family 31 member 1 (SLC31A1) mRNA expression data and clinicopathological characteristics in breast cancer were downloaded from the Cancer Genome Atlas (TCGA). The median expression of SLC31A1 was used to separate high/low expression groups. Human breast cancer tissues (*n* =  27) and normal breast tissues adjacent to the tumor (*n* =  27) were obtained from our breast center, Fujian Medical University Union Hospital. We perform prognostic analysis through the Kaplan–Meier Plotter website^1^.

### Functional enrichment analysis

**Figure 1:**
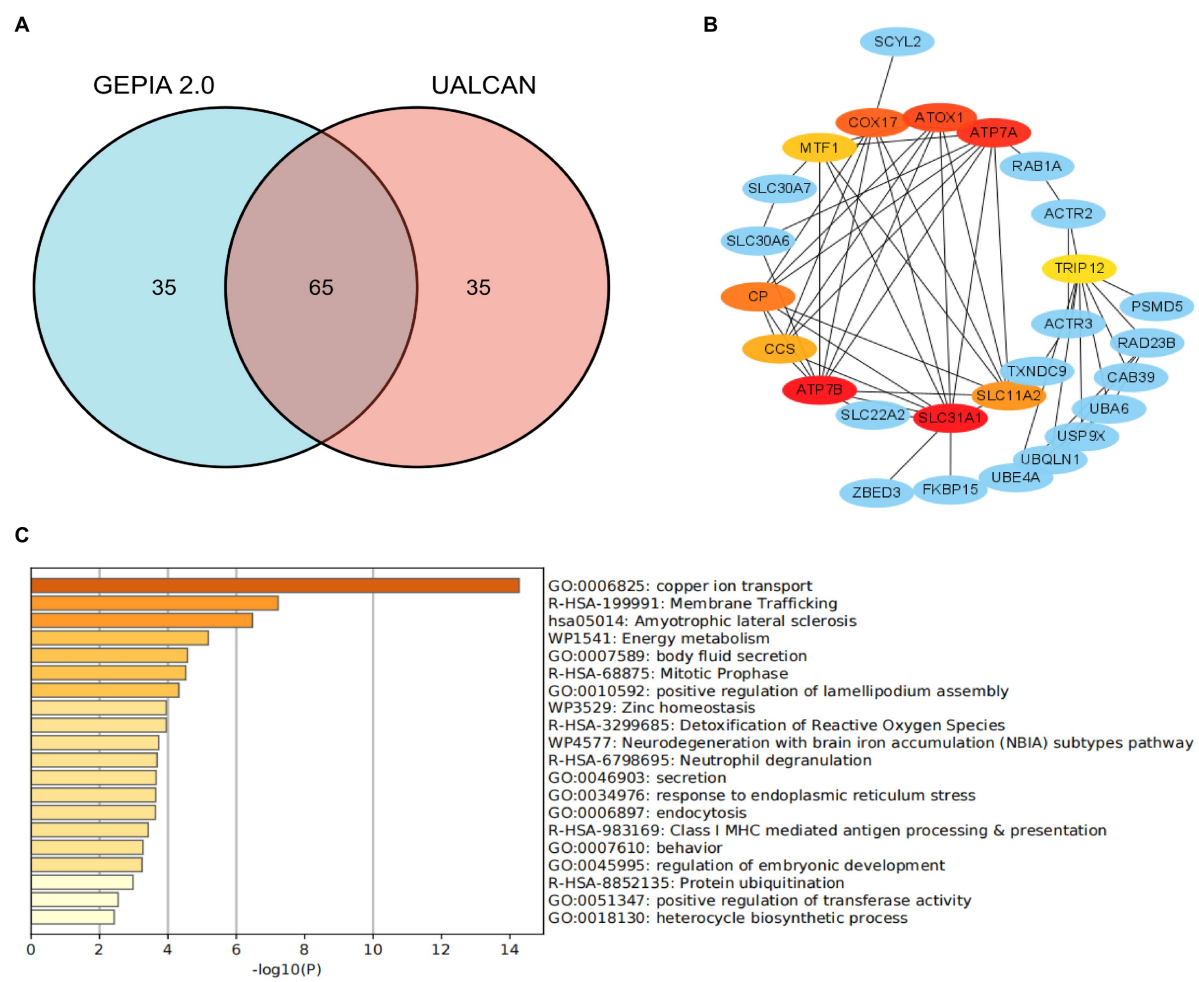
Co-expression network of SLC31A1 and functional enrichment analysis. **(A)** Venn diagram of the SLC31A1-related genes of breast cancer from GEPIA2.0 and UALCAN websites. **(B)** SLC31A1-related gene co-expressed network. **(C)** Functional enrichment analysis of co-expressed genes of SLC31A1.

STRINGS^2^ is an online website, which was used for protein–protein interaction (PPI) network analysis on SLC31A1. The result revealed that there were 10 proteins binding with SLC31A1, including ATP7B, MTF1, SLC22A2, ZBED3, CCS, CP, COX17, ATP7A, SLC11A2, and ATOX1. Furthermore, we, respectively, obtained the top 50 significant genes that are positively correlated and negatively correlated with SLC31A1 through the GEPIA2 website^3^ and the UALCAN website^4^. We took an intersection of 100 related genes between these two data sets and obtained 65 common genes (Figure 1A). Combined with 10 binding proteins and 65 related genes of SLC31A1, the functional enrichment analysis was performed by the Metascape website^5^.

### Relationship between the SLC31A1 expression and m6A-related genes

**Figure 2:**
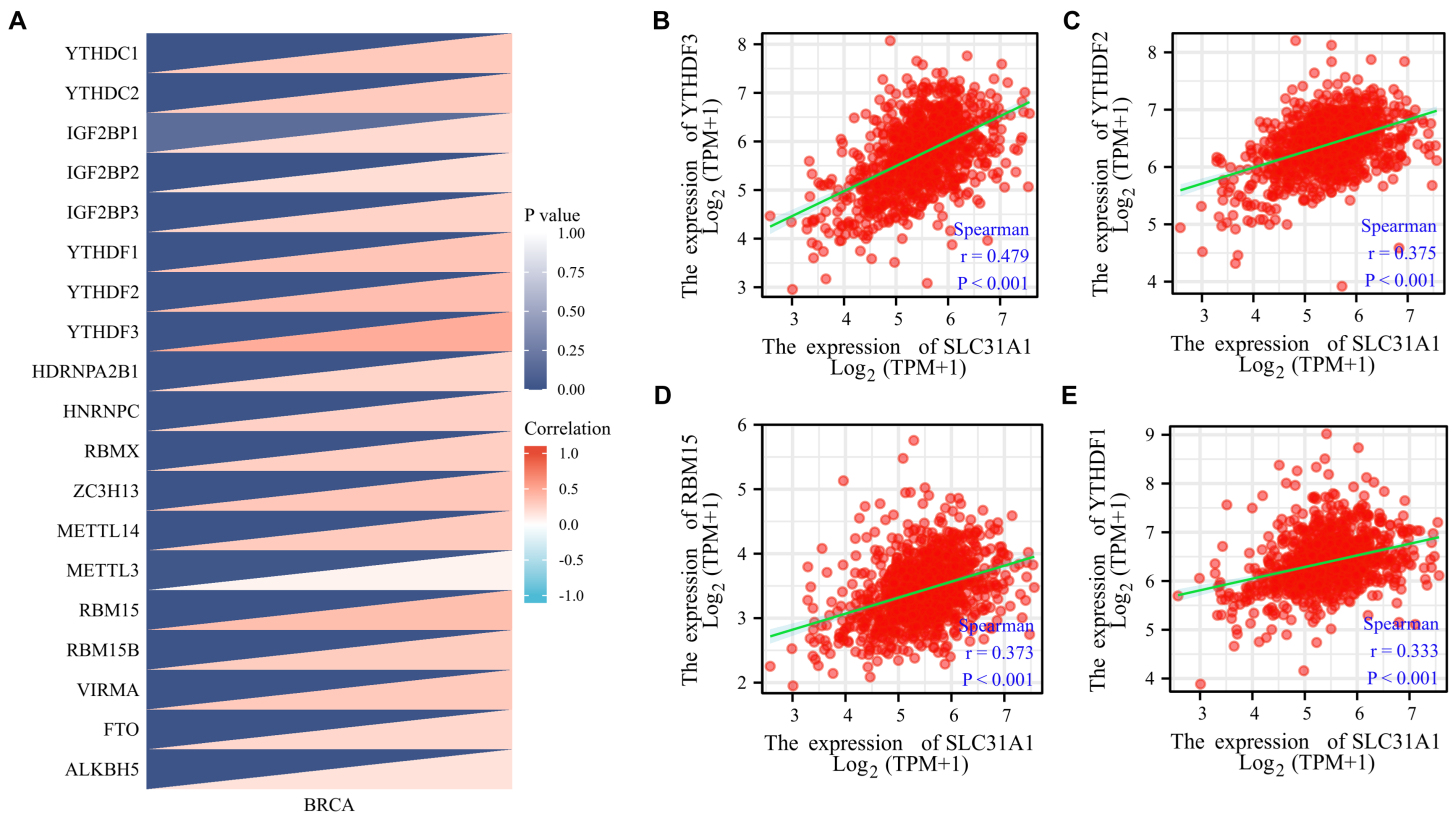
Correlations of the SLC31A1 expression with m6A-related genes in breast cancer based on the TCGA database. **(A)** Correlation between the expression level of SLC31A1 and m6A-related genes. **(B–E)** The expression of SLC31A1 was positively associated with YTHDF3 **(B)**, YTHDF2 **(C)**, RBM15 **(D)**, and YTHDF1 **(E)**. Spearman’s rank correlation coefficient was applied for correlation analysis.

Our study attempted to explore the relationship between the SLC31A1 expression and the expression of m6A-related genes in BRCA, and a correlation heat map was used to demonstrate them. All m6A-related genes analyzed are shown in Figure 2.

### Prediction of miRNA

The starBase website^6^ is an open-source platform, which was used for decoding miRNA–ceRNA and miRNA–ncRNA interaction networks in our study (10, 11). The website integrates seven databases, containing PITA, RNA22, miRmap, microT, miRanda, PicTar, and TargetScan, and was employed to forecast upstream miRNAs potentially binding to SLC31A1. The CLIP data of the website for this study was set as strict stringency (≥5), and the other basic settings as default. In this study, at least three database-predicted miRNAs were included in the analysis. Then, we established the miRNA-SLC31A1 regulatory network with Cytoscape software. In addition, we used this website for the correlation analysis between target lncRNA and miRNA.

### Prediction of lncRNA and ceRNA network construction

The website miRNet^7^ is an integrated open-source platform linking miRNAs from several miRNA-linked databases (TarBase, miRTarBase, miRecords, and miRanda). In this study, we predicted potential target lncRNAs of miRNA via this website. Subsequently, lncRNAs predicted by miRNet and starBase database were intersected to obtain the most potential regulatory lncRNAs. A comprehensive analysis of integrative miRNA–mRNA and miRNA–lncRNA was conducted with a negative correlation between expression levels to establish a key lncRNA–miRNA–mRNA (SLC31A1) ceRNA network for BRCA.

### Immune infiltration analysis

The Tumor Immune Estimation Resource (TIMER)^8^ was used for a comprehensive analysis of tumor-infiltrating immune cells in our study. Several researchers have suggested that tumor-infiltrating lymphocytes(TILs) were evaluated as a prognostic feature in multiple molecular subtypes of BRCA (12–14). Our study attempted to analyze the associations and prognosis between immune infiltrate cells (CD8+ T cells, CD4+ T cells, B cells, dendritic cells, macrophages, and neutrophils) and SLC31A1 expression by the TIMER website. In addition, we analyzed the correlation between the expression level of SLC31A1 in BRCA and the immune marker. A recent study has shown that cancer-associated fibroblast(CAF)plays a vital role in cancer progression and immunity (15). CAF is a stromal component that constitutes the tumor microenvironment (TME). To evaluate the role of SLC31A1 in the tumor microenvironment, we analyzed the correlation between the SLC31A1 expression and CAF in pan-cancer by the TIMER 2.0 website^9^.

### Cell lines culture

In this study, breast cancer cell lines (MCF-7 and BT-549) and the non-tumorigenic epithelial breast cell line (MCF-10A) were purchased from the Chinese Academy of Sciences (Shanghai, China) and cultured at 37°C with 5% CO_2_ in a humidified incubator. The DMEM medium (Gibco) with 10% fetal bovine serum (FBS; Gibco) was used to culture MCF-7 and BT-549 cell lines. DMEM/Ham’s F-12 (1,1; Gibco) with 5% FBS (Gibco) was used to culture MCF-10A.

### RNA extraction and quantitative real-time PCR

Total RNA was isolated from cells using Trizol (Takara, Dalian, China) and reverse transcribed by using the PrimeScriptTM RT reagent kit (TaKaRa). Real-time PCR(RT-qPCR)was performed using the SYBR Premix Ex Taq II kit (Takara). GAPDH was used as an internal reference, and the relative expression of SLC31A1 mRNAs was quantified using the 2 − ΔΔCt method. Primer sequences were:

SLC31A1 forward, 5’-GCTACTTCCTCATGCTCATCTTC-3′;
SLC31A1 reverse, 5’-TATCCACTACCACTGCCTTCTT-3′;
GAPDH forward, 5’-GTCTCCTCTGACTTCAACAGCG-3′; and.
GAPDH reverse, 5’-ACCACCCTGTTGCTGTAGCCAA-3′.

### Statistical analysis

In this study, the Xiantao Academic website^10^ was used to perform most of the statistical analysis. Furthermore, we also conducted mapping and statistical analysis through GraphPad Prism software (version 7.0). Spearman’s rank correlation coefficient was applied for correlation analysis. A value of p of <0.05 was considered statistically significant. The *t*-test was used to compute statistical significance and annotated by the number of stars (*, value of *p* <0.05; **, value of *p* <0.01; and ***, value of *p* <0.001).

## Results

### SLC31A1 was over-expressed in breast tumor tissues and cell lines

**Figure 3:**
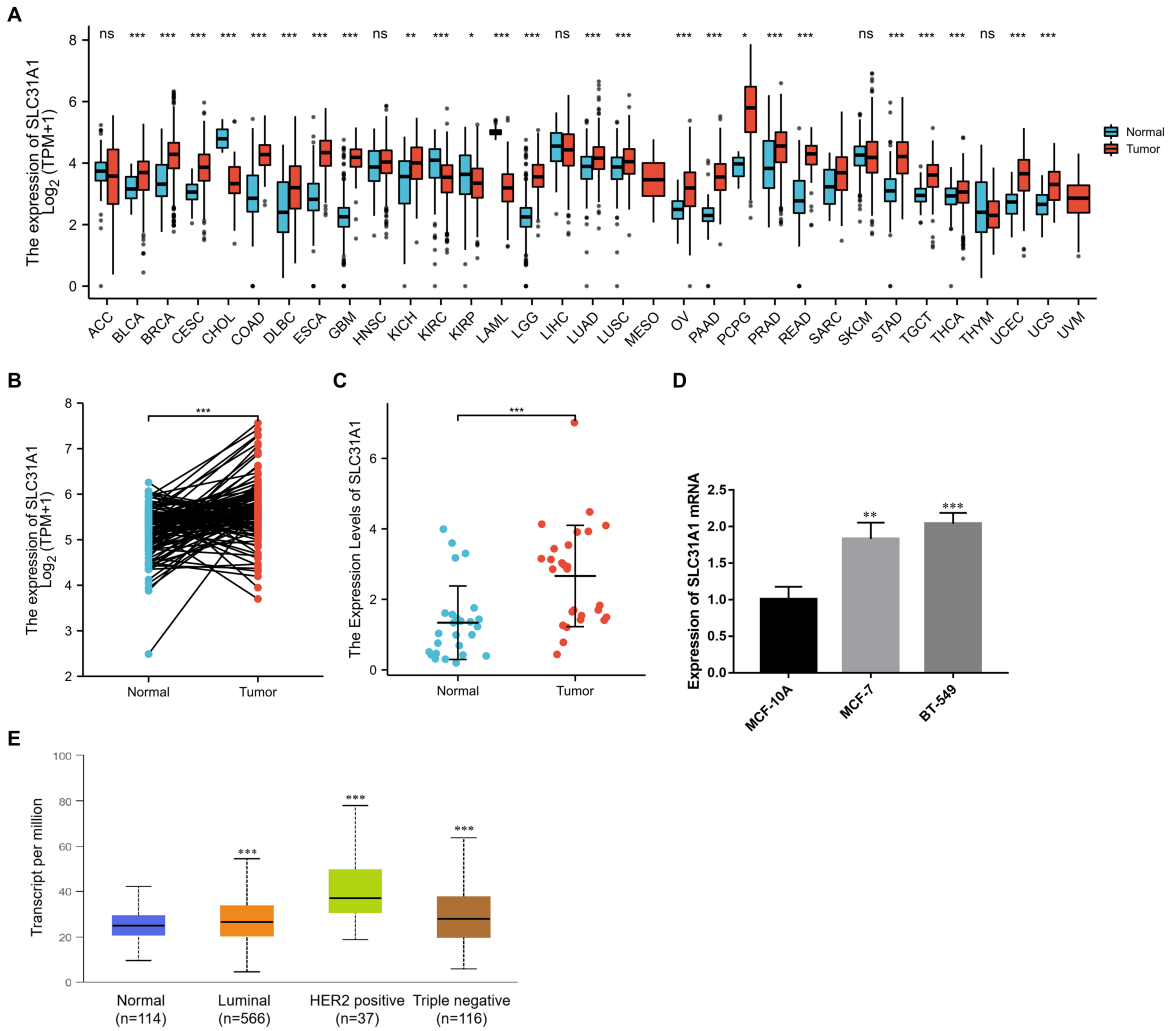
Differential expression of SLC31A1 in breast cancer (BRCA). **(A)** SLC31A1 mRNA expression in pan-cancer tissues compared to normal tissues based on the TCGA database. **(B)** SLC31A1 mRNA expression of tumor tissues in BRCA compared to pairwise normal tissues based on TCGA database. **(C)** The mRNA expression level of SLC31A1 is higher in breast cancer tissues (*n* = 27) than in paired normal breast tissues (*n* = 27). **(D)** SLC31A1 mRNA expression in different breast cancer cell lines (MCF-7 and BT-549) and human mammary epithelial cells (MCF-10A). **(E)** SLC31A1 mRNA expression in different breast cancer molecular subtypes. The *t*-test was used to compare the expression differences between the two groups. *p < 0.05, **p < 0.01, and ***p < 0.001.

To determine whether SLC31A1 plays a role in human cancers, we first examined the expression level of SLC31A1 in the TCGA database. Our result suggested that the expression of SLC31A1 mRNA was upregulated in 22 tumor types while downregulated in four tumor types compared with the normal tissue (Figure 3A). In a further pairwise comparison, SLC31A1 mRNA expression was also upregulated in BRCA (Figure 3B). Furthermore, we examined the expression of SLC31A1 using real-time PCR in human breast samples, breast tumor cell lines (MCF-7 and BT-549), and human mammary epithelial cells (MCF10A). The result revealed that the mRNA expression level of SLC31A1 was higher in breast cancer tissues than in normal breast tissues (Figure 3C). Similarly, the mRNA expression level of SLC31A1 was also higher in the luminal (MCF-7) cell line and triple-negative breast tumor cell line (BT-549) than in normal breast cell lines (MCF-10A) (Figure 3D). Furthermore, we found that the expression level of SLC31A1 in the tumor tissue of all breast cancer subtypes was higher than that in the normal tissue through the UALCAN website (Figure 3E).

### Correlation of SLC31A1 with clinicopathological characteristics and survival analysis

**Figure 4:**
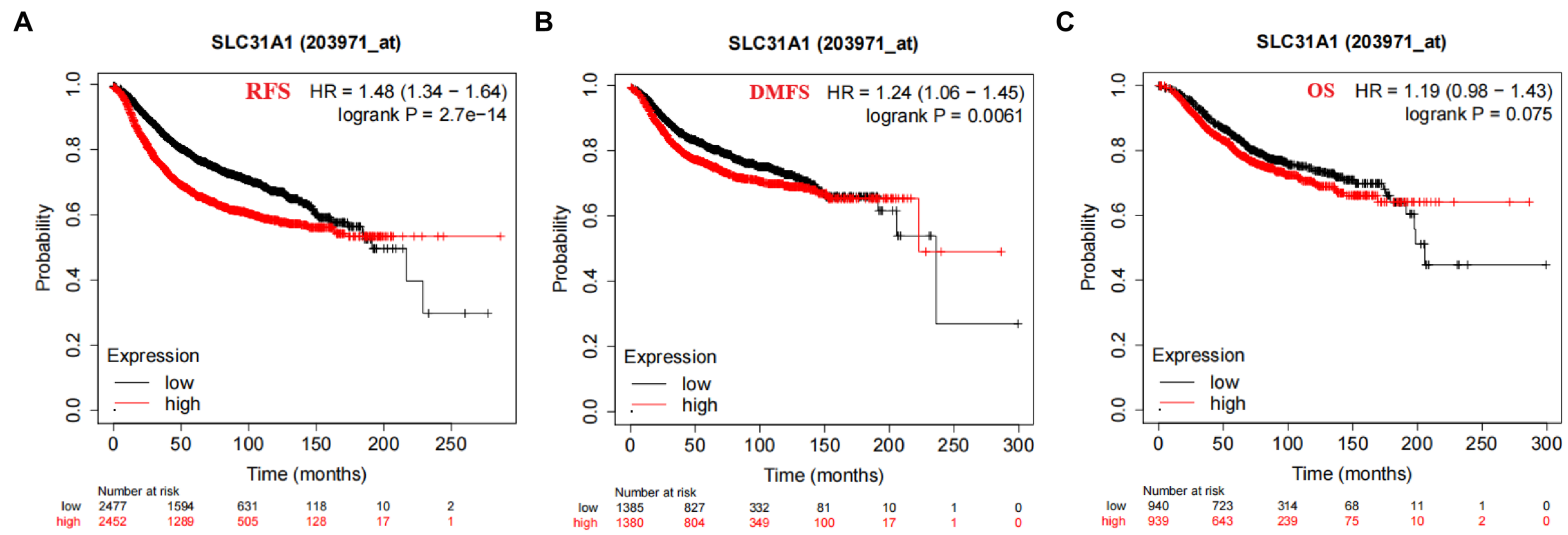
Correlation of SLC31A1 mRNA expression and survival in breast cancer from the Kaplan–Meier Plotter website. **(A)** The Kaplan–Meier curve of the relapse-free survival (RFS) probability in 203971_at breast cancer cohort. **(B)** The Kaplan–Meier curve of the distant metastasis-free survival (DMFS) probability in 203971_ at the breast cancer cohort. **(C)** The Kaplan–Meier curve of the overall survival (OS) probability in 203971_ at the breast cancer cohort.

In our study, the correlation between the SLC31A1 expression and clinicopathological characteristics in breast cancer patients based on the TCGA database was analyzed. As shown in Table 1, the differential expression of SLC31A1 was significantly related to race (*p* = 0.004), ER status (*p* < 0.001), HER2 status (*p* = 0.026), and PAM50 (*p* < 0.001). Furthermore, the Kaplan–Meier survival analysis showed that the high expression of SLC31A1 in breast cancer patients was related to the worse relapse-free survival (RFS) (HR = 1.48, *p* = 2.7e-14) (Figure 4A), distant metastasis-free survival (DMFS) (HR = 1.24, *p* = 0.0061) (Figure 4B), and worse overall survival (OS) trend (HR = 1.19, *p* = 0.075) (Figure 4C).

**Table 1:** Correlation of SLC31A1 with clinicopathological characteristics in The Cancer Genome Atlas (TCGA) cohort.

Characteristic	Low expression of SLC31A1	High expression of SLC31A1	*p*
N (%)	541	542	
**Age**			0.739
<=60	297 (27.4%)	304 (28.1%)	
>60	244 (22.5%)	238 (22%)	
**Race**			0.004
Asian	26 (2.6%)	34 (3.4%)	
Black or African American	112 (11.3%)	69 (6.9%)	
White	370 (37.2%)	383 (38.5%)	
**T stage**			0.610
T1	140 (13%)	137 (12.7%)	
T2	312 (28.9%)	317 (29.4%)	
T3	73 (6.8%)	66 (6.1%)	
T4	14 (1.3%)	21 (1.9%)	
**N stage**			0.251
N0	266 (25%)	248 (23.3%)	
N1	179 (16.8%)	179 (16.8%)	
N2	60 (5.6%)	56 (5.3%)	
N3	30 (2.8%)	46 (4.3%)	
**M stage**			0.616
M0	435 (47.2%)	467 (50.7%)	
M1	8 (0.9%)	12 (1.3%)	
**Pathologic stage**			0.769
Stage I	97 (9.2%)	84 (7.9%)	
Stage II	308 (29.1%)	311 (29.3%)	
Stage III	124 (11.7%)	118 (11.1%)	
Stage IV	8 (0.8%)	10 (0.9%)	
**ER status**			< 0.001
Negative	94 (9.1%)	146 (14.1%)	
Indeterminate	1 (0.1%)	1 (0.1%)	
Positive	421 (40.7%)	372 (35.9%)	
**PR status**			0.062
Negative	155 (15%)	187 (18.1%)	
Indeterminate	3 (0.3%)	1 (0.1%)	
Positive	358 (34.6%)	330 (31.9%)	
**HER2 status**			0.026
Negative	276 (38%)	282 (38.8%)	
Indeterminate	8 (1.1%)	4 (0.6%)	
Positive	61 (8.4%)	96 (13.2%)	
**PAM50**			< 0.001
Normal	17 (1.6%)	23 (2.1%)	
LumA	320 (29.5%)	242 (22.3%)	
LumB	94 (8.7%)	110 (10.2%)	
Her2	21 (1.9%)	61 (5.6%)	
Basal	89 (8.2%)	106 (9.8%)	

### Co-expression network of SLC31A1 and functional enrichment analysis

As shown in Figure 1B, we constructed a co-expression network with 10 interacting proteins and 65 common co-expressed genes. Functional enrichment analysis indicated that these genes were significantly related to copper ion transport, body fluid secretion, positive regulation of lamellipodium assembly, and so on (Figure 1C). These results suggested that SLC31A1 interaction with these proteins may affect the transfer of copper ions and induce cuproptosis.

### The SLC31A1 expression and m6A RNA methylation-related genes

More recently, m6A modifications are associated with breast cancer progression (16, 17). To further assess whether SLC31A1 is related to m6A modification, we assessed the association between the expression of SLC31A1 and 20 m6A-related genes in breast cancer. Results revealed that the SLC31A1 expression was significantly positively associated with all m6A-related genes in breast cancer (Figure 2A). Importantly, the SLC31A1 expression was strongly positively associated with YTHDF3 (r = 0.479, *p* < 0.001), YTHDF2 (*r* = 0.375, *p* < 0.001), RBM15 (*r* = 0.373, *p* < 0.001), and YTHDF1 (*r* = 0.333, *p* < 0.001) (Figures 2B–E).

### Predicted upstream potential miRNA of SLC31A1

**Figure 5:**
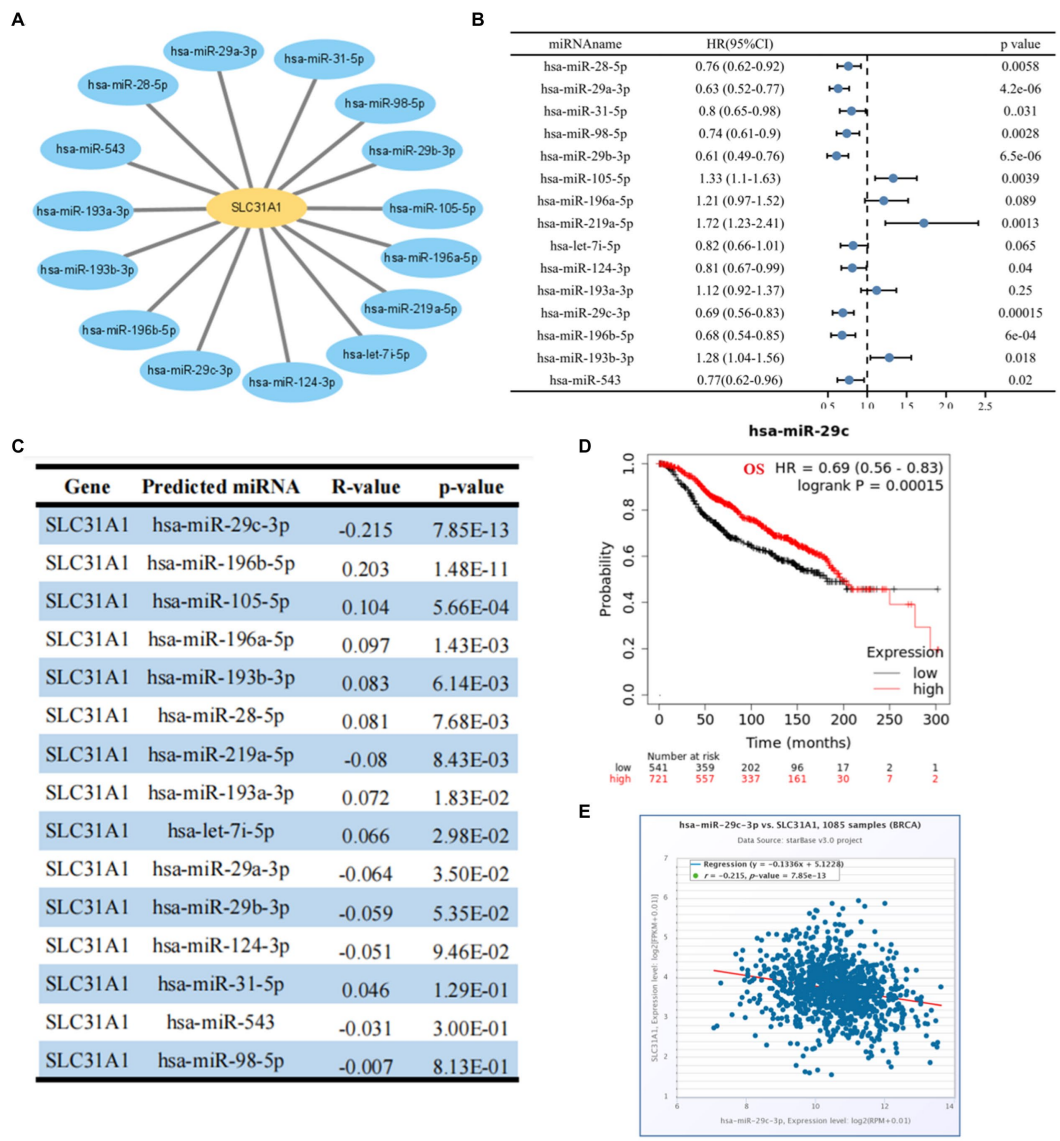
Identification of upstream potential miRNA of SLC31A1 in breast cancer. **(A)** miRNA-SLC31A1 network constructed by Cytoscape software. **(B)** Prognostic analysis (overall survival, OS) of potential upstream miRNAs of SLC31A1 in breast cancer using forest plots. **(C)** Expression correlation between predicted miRNAs and SLC31A1 in breast cancer using starBase database. **(D)** Overall survival (OS) analysis for hsa-miR-29c-3p in breast cancer. **(E)** Scatter plot about the correlation between the expression of SLC31A1 and hsa-miR-29c-3p in breast cancer obtained from the starBase database.

Many studies have proven that miRNA plays its biological function by participating in the regulation of the translation process of its downstream genes. A recent study showed that an ATPase copper transporter promotes breast cancer cells’ cisplatin resistance by being negatively regulated by miR-148a-3p (18). Therefore, our study attempted to predict the upstream regulatory miRNA genes of SLC31A-the copper death-related gene. As shown in Figure 5A, we finally screened out 15 predicted miRNAs that could bind to SLC31A1 through the starBase website. Furthermore, we performed a prognostic analysis of predicted miRNAs and an expression correlation analysis between miRNA and SLC31A1. Hsa-miR-28-5p, hsa-miR-29a-3p, hsa-miR-31-5p, hsa-miR-98-5p, hsa-miR-29b-3p, hsa-miR-124-3p, hsa-miR-29c-3p (Figure 5D), hsa-miR-196b-5p, and hsa-miR-543 were favorable prognostic biomarkers for OS of patients with breast cancer, while hsa-miR-105-5p, hsa-miR-219a-5p, and hsa-miR-193b-5p were unfavorable prognostic biomarkers (Figure 5B). The expression correlation between predicted miRNAs and SLC31A1 in breast cancer using the starBase database is shown in Figure 5C and Supplementary Table S1. As shown in Figure 5E, hsa-miR-29c-3p had the strongest negative correlation with SLC31A1 expression. Combined with these results, hsa-miR-29c-3p was identified to be the most potential miRNA regulating SLC31A1 in breast cancer.

### LINC00511-Mir-29c-3p-SLC31A1 axis is a potential pathway promoting breast cancer progress by affecting copper transport

**Figure 6:**
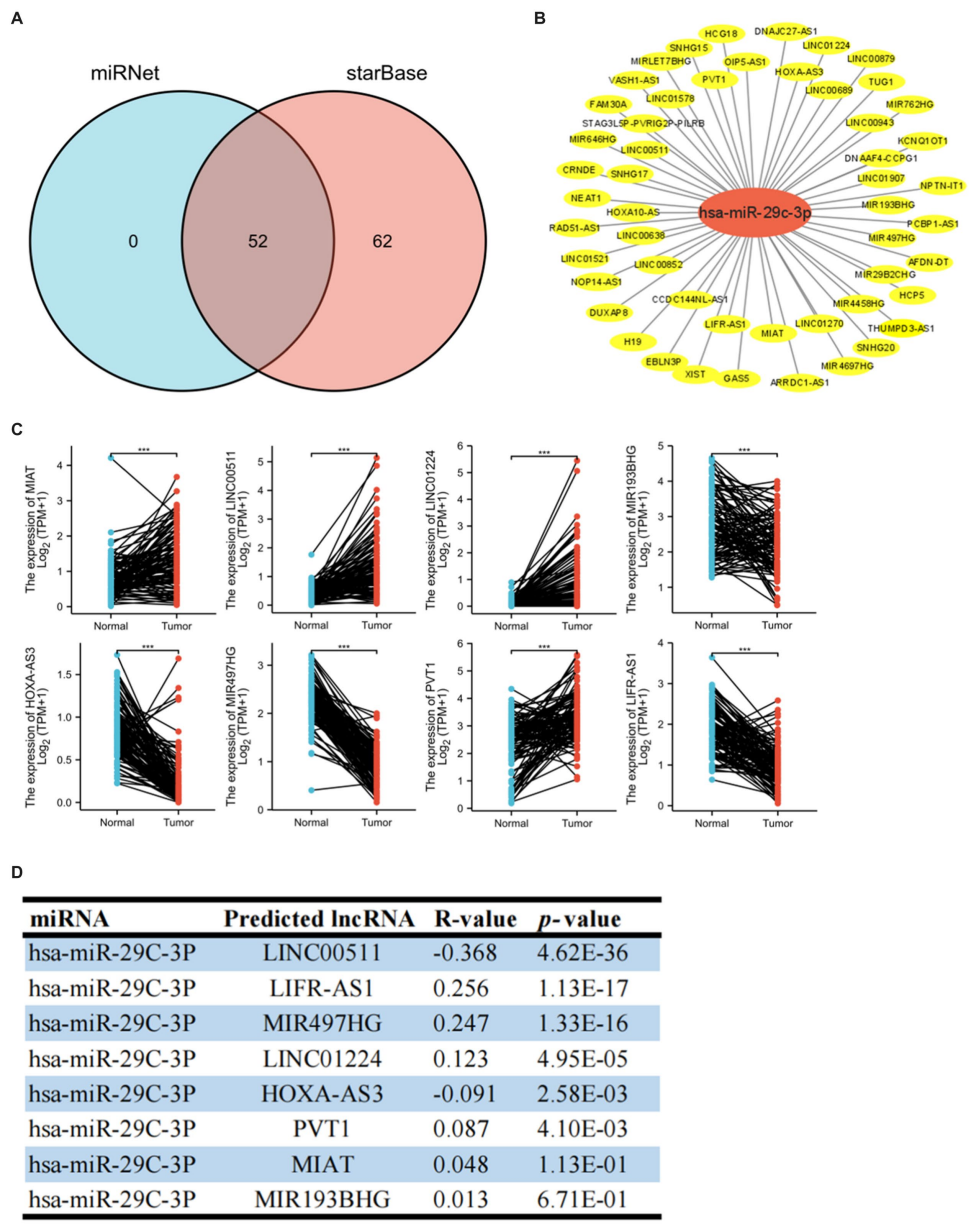
Identification of upstream potential lncRNA of hsa-miR-29c-3p in breast cancer. **(A)** Venn diagram of upstream potential lncRNA of hsa-miR-29c-3p from miRNet and starBase websites. **(B)** lncRNA-hsa-miR-29c-3p network constructed by Cytoscape software. **(C)** Eight of the 52 predicted lncRNAs are differentially expressed in breast cancer based on the TCGA database. **(D)** Expression correlation in breast cancer between predicted lncRNAs and hsa-miR-29c-3p using starBase database. The *t*-test was used to compare the expression differences between the two groups. *p < 0.05, **p < 0.01, ***p < 0.001.

A growing body of evidence has revealed that lncRNA promotes breast cancer progression and functions as ceRNA to target genes by affecting mi-RNA (6, 19, 20). Therefore, we predicted potential lncRNAs that might bind to hsa-miR-29c-3p by starBase and miRNet websites and then took the intersection (Figure 6A). We finally found 52 common predicted lncRNAs, and then the lncRNA-hsa-miR-29c-3p regulatory network was constructed using Cytoscape software (Figure 6B). The TCGA database was used to determine whether these lncRNAs were differentially expressed in breast cancer tissues compared to normal tissues. As shown in Figure 6C, a total of eight lncRNAs were significantly downregulated or upregulated in breast cancer compared to the normal tissues. The prognostic value of these eight lncRNAs in breast cancer was then evaluated. However, all eight lncRNAs show no statistically significant difference in survival(data not shown). Subsequently, the starBase database was used to evaluate the expression correlation between these eight lncRNAs and hsa-miR-29c-3p. The strongest negative expression correlation between LINC00511 and hsa-miR-29c-3p (*R* = −0.368, *p* = 4.62e-36) is shown in Figure 6D and Supplementary Table S2. Combined with the expression analysis, survival analysis, and correlation analysis, LINC00511 was considered the most significant lncRNA upstream of the hsa-miR-29c-3p/SLC31A1 axis in breast cancer.

### Positive correlation between the SLC31A1 expression and tumor immune infiltration

**Figure 7:**
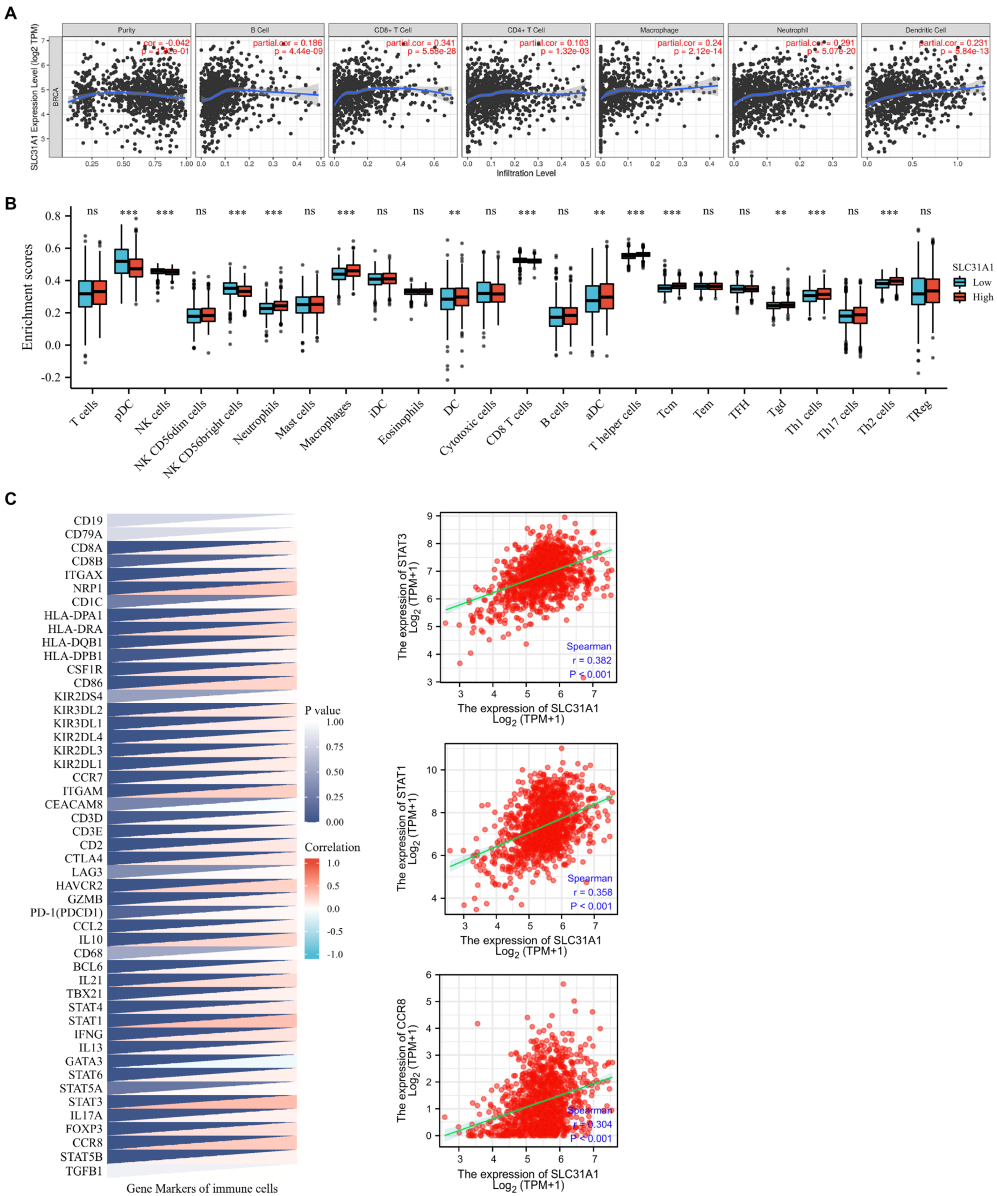
Correlations of the SLC31A1 expression with immune cells infiltration and immune markers in breast cancer. **(A)** The expression of SLC31A1 was significantly correlated with infiltrating levels of B cells, CD8 + T cells, CD4 + T cells, macrophages, neutrophils, and dendritic cells in breast cancer. The SLC31A1 expression levels against tumor purity adjustment are displayed on the left-most panel. **(B)** The difference in enrichment scores of 24 immune cell types between the SLC31A1 high and low expression groups. **(C)** Correlation between SLC31A1 and immune markers of various tumor immune infiltration cells in breast cancer. The *t*-test is used to compare the expression differences between the two groups. *p < 0.05, **p < 0.01, ***p < 0.001.

A recent study showed that intratumoral and stromal CD4+ T cell density is an independent predictor of pCR in triple-negative patients (21). Our study attempted to investigate whether the expression of SLC31A1 is related to the level of immune infiltration in breast cancer through the TIMER website. The SLC31A1 expression showed a positive correlation with the levels of B cells (cor = 0.186, *p* = 4.44e-6), CD8+ T cells (cor = 0.341, *p* = 5.58e-28), CD4 + T cells (cor = 0.103, *p* = 1.32e-3), macrophages (cor = 0.24, *p* = 2.12e-14), neutrophils (cor = 0.291, *p* = 5.07e-20), and dendritic cells (DC, cor = 0.231, *p* = 5.84e-13) (Figure 7A). B cells, macrophages, and dendritic cells have been reported to be associated with tumor immune escape. Therefore, SLC31A1 may positively regulate B cells, macrophages, and dendritic cells to promote tumor immune escape. Furthermore, we analyzed the differential expression of 24 immune cells between the high SLC31A1 expression group and the low SLC31A1 expression group in breast cancer. Compared with the low expression group, aDC, DC, iDC, macrophage, neutrophil, T helper cells, Tcm, Tgd, Th1, and Th2 increased in the high expression group of SLC31A1, while CD8 + T cell, NK CD56bright cells, and pDC decreased (Figure 7B). In addition, we explored the correlation between SLC31A1 and immune markers of various tumor immune infiltration cells in breast cancer. The results showed that the expression of SLC31A1 was significantly positively correlated with immune markers in breast cancer, especially for STAT3 of Th17 (*r* = 0.382, *p* < 0.001), STAT1 of Th1 (*r* = 0.358, *p* < 0.001), and CCR8 of Treg (*r* = 0.304, *p* < 0.001) (Figure 7C).

**Figure 8:**
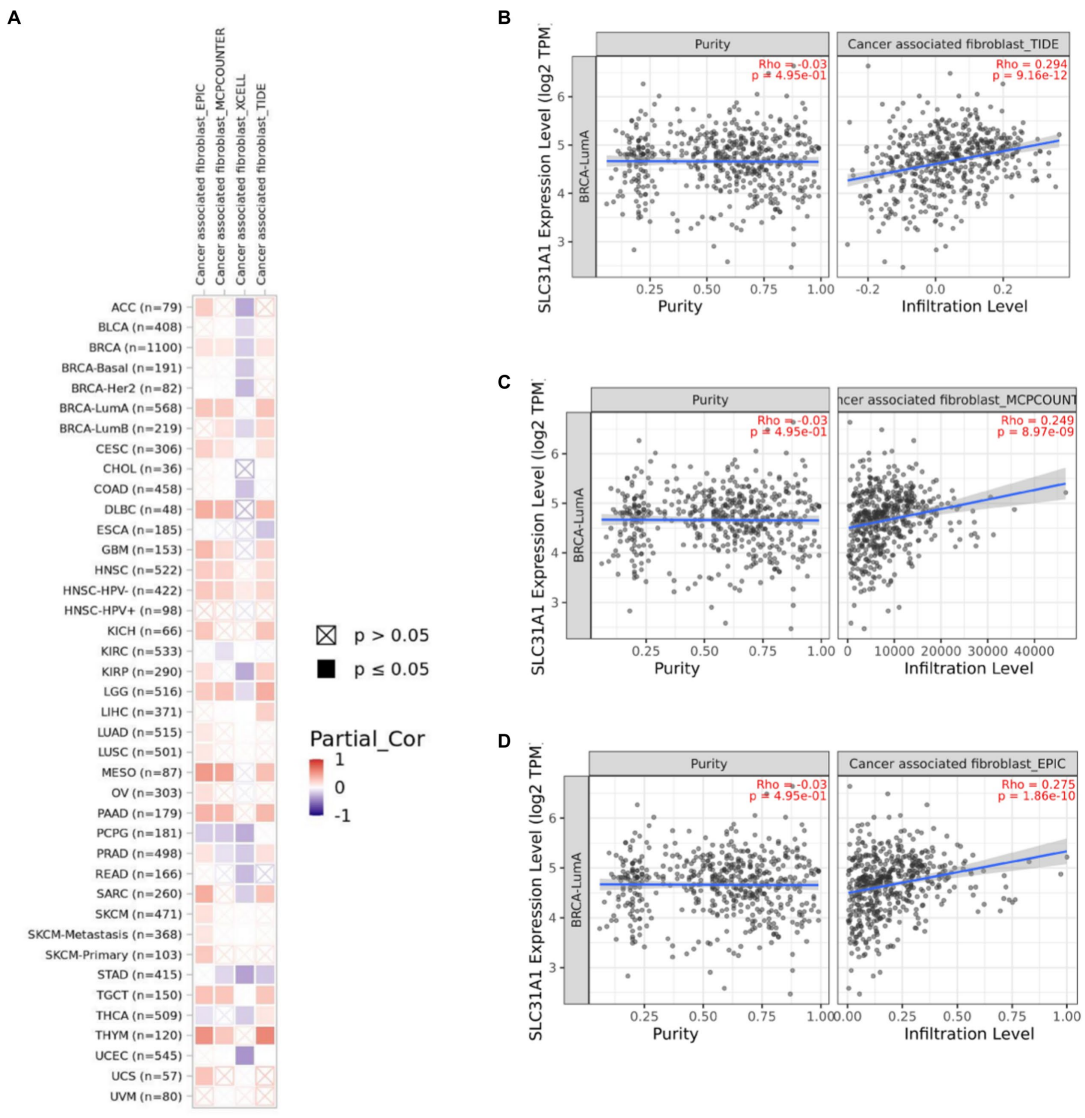
Correlation of the SLC31A1 expression and cancer-associated fibroblast(CAF)by TIMER 2.0 website. **(A)** Correlation of the SLC31A1 expression and CAF in pan-cancers. **(B)** The SLC31A1 expression in the BRCA-LumA subtype was positively associated with CAF by the TIDE algorithm. **(C)** The SLC31A1 expression in the BRCA-LumA subtype was positively associated with CAF by the MCPCOUNTER algorithm. **(D)** The SLC31A1 expression in the BRCA-LumA subtype was positively associated with the CAF by EPIC algorithm. *The SLC31A1 expression levels against tumor purity adjustment were displayed on the left panel.

Several studies have shown that CAF plays an essential role in TME and correlates with resistance and tumor progression (22–24). Therefore, we analyzed the correlation between the SLC31A1 expression and CAF by the TIMER 2.0 website. We found that the SLC31A1 expression was positively related to CAF in most tumors (Figure 8A). For BRCA-LumA disease, the SLC31A1 expression was positively associated with CAF by TIDE (Figure 8B), MCPCOUNTER (Figure 8C), and EPIC (Figure 8D) algorithms on the TIMER 2.0 website.

## Discussion

Copper is an essential metal nutrient for normal physiology. It has been demonstrated that enhanced copper levels in tumors lead to cancer progression (25). As the copper importer, SLC31A1 was identified as an important regulatory gene in copper death. Research has shown that the SLC31A1 expression level was correlated with the malignant degree of pancreatic cancer. However, the expression level and function of SLC31A1 in breast cancer are still unclear.

This study analyzed the expression and prognostic value of SLC31A1 in breast cancer and constructed a ceRNA regulatory network. We found that the expression of SLC31A1 mRNA was upregulated in breast cancer tissues and breast cancer cell lines. Further subgroup analysis showed that the expression level of SLC31A1 in breast cancer tissues of all subtypes (luminal, HER2 positive, and triple negative) was higher than that in normal breast tissues. In addition, high expression of SLC31A1 was associated with worse RFS and DMFS. The study by Rachel et al. showed that the expression of SLC31A1 is closely related to the efficacy of platinum-based anticancer regimens (26). Furthermore, we constructed a co-expression network of SLC31A1 and performed functional enrichment analysis, which is mainly responsible for the transport of copper ions. As shown in Figure 1B, ATP7A and ATP7B were the most important interacting proteins of SLC31A1. Previous studies have shown that ATPases-ATP7A and ATP7B serve as the major copper exporters and synergize with SLC31A1 maintaining copper homeostasis (27). Yu et al. found that copper deficiency makes pancreatic cancer cells dormant and leads to increased autophagy to resist the death of pancreatic cancer cells (5).

As is known, one of the most abundant posttranscriptional modifications in eukaryotic mRNA is m6A methylation. Yu et al. have reported that m6A “writer” METTL3 can downregulate COL3A1 expression by increasing its m6A methylation to inhibit TNBC cell metastasis (28). Our study suggested that the expression of SLC31A1 is strongly positively associated with YTHDF3, YTHDF2, RBM15, and YTHDF1. Chang et al. found that m6A “reader” YTHDF3 over-expression enhances the translation of m6A-enriched transcripts for ST6GALNAC5, GJA1, and EGFR, promoting brain metastases in breast cancer patients (29). A recent study from Ramesh et al. also showed that YTHDF3/ZEB1 axis plays an important role in the progression and metastasis of TNBC (30). However, there is no relevant study to prove the regulatory relationship between YTHDF3 and SLC31A1. Combined with our correlation analysis, YTHDF3 may upregulate the expression of SLC31A1 by increasing its m6A methylation, promoting the metastasis of breast cancer cells.

**Figure 9:**
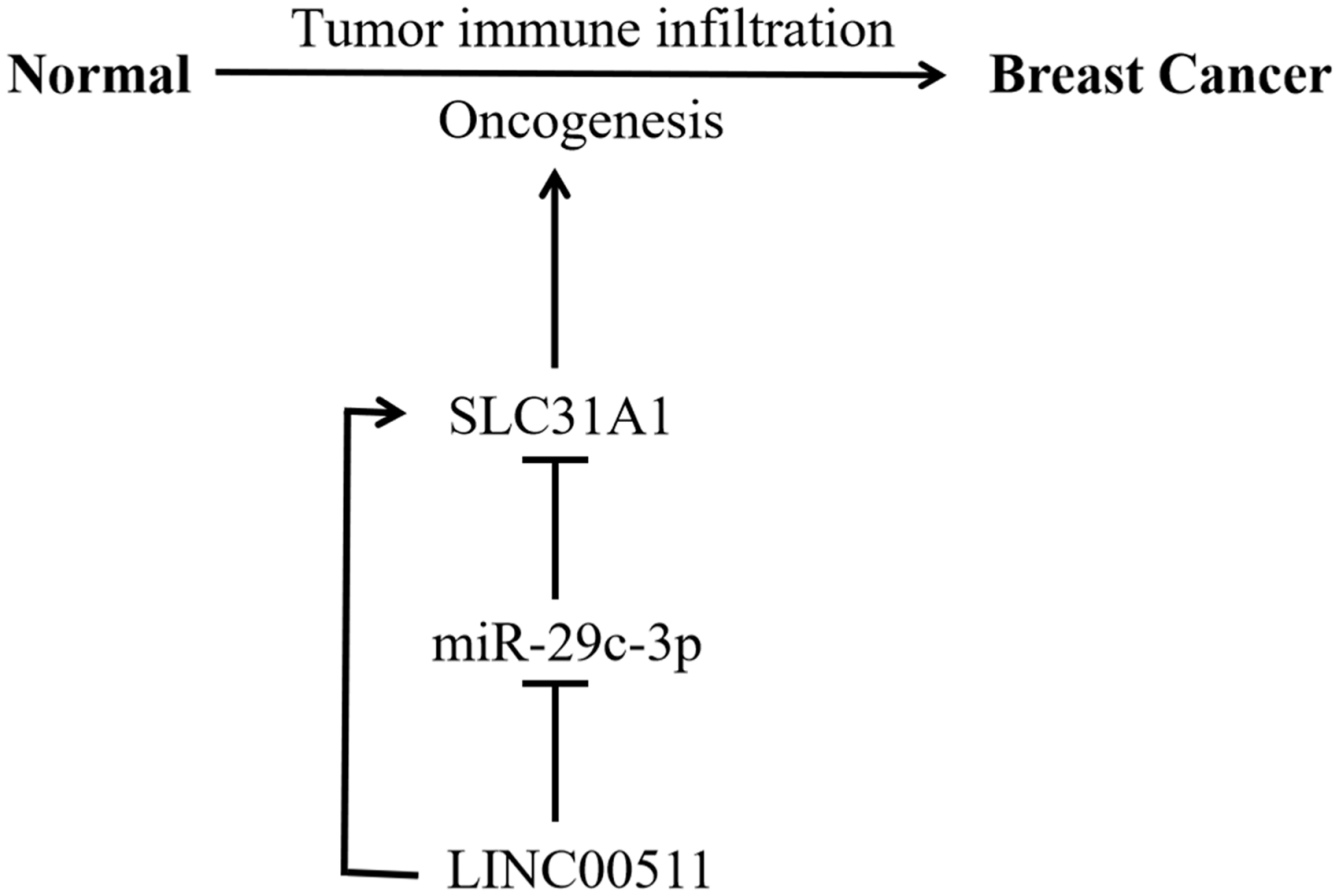
Model of LINC00511-miR-29c-3p-SLC31A1 axis in carcinogenesis of breast cancer.

MicroRNAs (miRNAs) are a class of evolutionarily highly conserved small non-coding RNAs and primarily affect gene expression levels *via* targeting mRNA (31). Growing research suggested that miRNAs contribute to cancer progression by regulating target genes (32–34). Our study supported that miR-29c-3p is the most potentially binding miRNA of SLC31A1. Correlation analysis suggested a strongly negative relationship between SLC31A1 and hsa-miR-29c-3p. The hsa-miR-29c-3p-SLC31A1 axis was considered as the potential pathway involved in the progression of breast cancer by regulating copper transport. Kong et al. suggested that lncRNA-CDC6 promotes breast cancer progression and functions as ceRNA to target CDC6 by sponging microRNA-215 (20). A recent study from Lou et al. showed that RP11-480I12.5–004 promoted growth and tumorigenesis of breast cancer by competitively binding to miR-29c-3p (35). These studies suggested that lncRNA serves as a ceRNA to regulate the expression of target genes in cancer *via* sponging miRNA. Our study further predicted the upstream potential lncRNAs of hsa-miR-29c-3p. As shown in Figure 6F analysis, LINC00511 was considered the most potential lncRNA upstream of hsa-miR-29c-3p. Through comprehensive analysis of lncRNA, miRNA, and mRNA expression, we constructed a ceRNA network and named it the LINC00511/miR-29c-3p/SLC31A1 axis (Figure 9). The LINC00511/miR-29c-3p/SLC31A1 axis may promote invasion and metastasis in breast cancer through the regulation of copper transport.

A deep analysis based on TCGA and tissue microarrays showed a strong correlation between the expression of SLC31A1 and PD-L1 in many cancers but not in normal tissues. Florida et al. found that adequate copper can increase the expression of PD-1/PD-L1 in cancer cells, while a copper deficiency can be linked to PD-L1-driven cancer immune escape (36). Moreover, the addition of copper-chelating drugs significantly increased the number of CD8 + T and natural killer cells and slowed down the growth of tumors in mice (36). These results suggest that SLC31A1 is closely related to tumor immune infiltration. Our results suggest that SLC31A1 was closely and positively associated with these major immune-infiltrating cells, especially with CD8 + T cells. Furthermore, we found that SLC31A1 was highly expressed in many immune cells in breast cancer, including aDC, DC, iDC, macrophage, neutrophil, T helper cells, Tcm, Tgd, Th1, and Th2. At present, a variety of immunocheckpoint inhibitors targeting PD-1 or PDL-1 have been approved by FDA for the treatment of breast cancer. Therefore, we further analyzed the relationship between SLC31A1 and immune checkpoints. The results revealed that the upregulation of the SLC31A1 expression is significantly correlated with STAT3 and STAT1. A study by Sasidharan et al. showed that dual inhibition of STAT1 and STAT3 activation could downregulate the expression of PD-L1 in human breast cancer cells (37). Florida et al. have reported that copper chelators inhibited the phosphorylation of STAT3 and promoted ubiquitin-mediated degradation of PD-L1 (36). These results suggested that SLC31A1 may affect STAT3 and STAT1 phosphorylation by regulating the concentration of copper ions in cells and then affecting the expression of PDL1.

CAF served as the predominant stromal cell type in the breast tumor microenvironment. Wen et al. have found that CAF-secreted IL32 promotes breast cancer cell invasion and metastasis *via* integrinβ3-p38 MAPK signaling (38). Research from Gao et al. indicated that CD63 + CAF induces tamoxifen resistance in breast cancer *via* exosomal miR-22 (39). These results showed that CAF plays a tumor promotion role in breast cancer. Moreover, we found that the expression of SLC31A1 was positively correlated with CAF in luminal A breast cancer (Figure 8). However, there is no report on SLC31A1 and CAF in breast cancer. Based on our analysis, future studies may focus on the underlining mechanism of SLC31A1 regulating CAF in luminal A breast cancer.

## Conclusion

In summary, SLC31A1 mRNA is upregulated in breast cancer and correlated with unfavorable prognosis. The expression level of SLC31A1 was positively correlated with tumor immune infiltration. We identified the LINC00511/miR-29c-3p axis as the upstream regulatory mechanism of SLC31A1 in breast cancer.

## Data availability statement

The datasets generated and analyzed during the current study are available in the persistent web link presented in the material and methods. All the persistent web links are given at: Kaplan–Meier Plotter (http://kmplot.com/analysis/index.php?p=service), STRINGS (https://string-db.org/, version: 11.5), GEPIA2 (http://gepia2.cancer-pku.cn/#index), UALCAN (http://ualcan.path.uab.edu/analysis-prot.html), Metascape (https://metascape.org/gp/index.html#/main/step1), starBase (www.starbase.sysu.edu.cn), miRNet (https://www.mirnet.ca/), TIMER (https://cistrome.shinyapps.io/timer/), TIMER 2.0 (http://timer.cistrome.org/), and Xiantao (https://www.xiantao.love/products/apply/c0b6febb-52dd-4525-970a-61bbe9e263ff/collect).

## Ethics statement

The studies involving human participants were reviewed and approved by the Ethics Committee of Fujian Medical University Union Hospital. The patients/participants provided their written informed consent to participate in this study.

## Author contributions

WL, DC, and CW designed the overall study and discussed and edited the manuscript. PY and LL contributed to the data analysis. WL drafted the manuscript and prepared all the figures and tables. All authors read and approved the manuscript.

## Funding

This study was supported by the Natural Science Foundation of Fujian Province, China (Grant No. 2020J011275) and the Quanzhou City Science and Technology Programme of China (Grant No. 2020N07319S).

## Conflict of interest

The authors declare that the research was conducted in the absence of any commercial or financial relationships that could be construed as a potential conflict of interest.

## Publisher’s note

All claims expressed in this article are solely those of the authors and do not necessarily represent those of their affiliated organizations, or those of the publisher, the editors and the reviewers. Any product that may be evaluated in this article, or claim that may be made by its manufacturer, is not guaranteed or endorsed by the publisher.

## Footnotes

^1^ http://kmplot.com/analysis/index.php?p=service

^2^https://string-db.org/, version: 11.5.

^3^ http://gepia2.cancer-pku.cn/#index

^4^ http://ualcan.path.uab.edu/analysis-prot.html

^5^ https://metascape.org/gp/index.html#/main/step1

^6^ www.starbase.sysu.edu.cn

^7^ https://www.mirnet.ca/

^8^ https://cistrome.shinyapps.io/timer/

^9^ http://timer.cistrome.org/

^10^ https://www.xiantao.love/products/apply/c0b6febb-52dd-4525-970a-61bbe9e263ff/collect
