# Supplementary material for: A ceRNA network-mediated over-expression of cuproptosis-related gene SLC31A1 correlates with poor prognosis and positive immune infiltration in breast cancer
Source: Front Med (Lausanne). 2023 May 18;10:1194046. doi: 10.3389/fmed.2023.1194046 (PMC10234574; doi:10.3389/fmed.2023.1194046)
Supplement: Supplementary file 1 [file Table_1.docx]

| **Supplement table 1.** The expression correlation between predicted miRNAs and SLC31A1 in breast cancer using starBase database. | | | |
| --- | --- | --- | --- |
| **Gene** | **Predicted miRNA** | **R-value** | **p-value** |
| SLC31A1 | hsa-miR-29c-3p | -0.215 | 7.85E-13 |
| SLC31A1 | hsa-miR-196b-5p | 0.203 | 1.48E-11 |
| SLC31A1 | hsa-miR-105-5p | 0.104 | 5.66E-04 |
| SLC31A1 | hsa-miR-196a-5p | 0.097 | 1.43E-03 |
| SLC31A1 | hsa-miR-193b-3p | 0.083 | 6.14E-03 |
| SLC31A1 | hsa-miR-28-5p | 0.081 | 7.68E-03 |
| SLC31A1 | hsa-miR-219a-5p | -0.08 | 8.43E-03 |
| SLC31A1 | hsa-miR-193a-3p | 0.072 | 1.83E-02 |
| SLC31A1 | hsa-let-7i-5p | 0.066 | 2.98E-02 |
| SLC31A1 | hsa-miR-29a-3p | -0.064 | 3.50E-02 |
| SLC31A1 | hsa-miR-29b-3p | -0.059 | 5.35E-02 |
| SLC31A1 | hsa-miR-124-3p | -0.051 | 9.46E-02 |
| SLC31A1 | hsa-miR-31-5p | 0.046 | 1.29E-01 |
| SLC31A1 | hsa-miR-543 | -0.031 | 3.00E-01 |
| SLC31A1 | hsa-miR-98-5p | -0.007 | 8.13E-01 |
