# Supplementary material for: A ceRNA network-mediated over-expression of cuproptosis-related gene SLC31A1 correlates with poor prognosis and positive immune infiltration in breast cancer
Source: Front Med (Lausanne). 2023 May 18;10:1194046. doi: 10.3389/fmed.2023.1194046 (PMC10234574; doi:10.3389/fmed.2023.1194046)
Supplement: Supplementary file 2 [file Table_2.docx]

| **Supplement table 2.** The expression correlation in breast cancer between predicted lncRNAs and hsa-miR-29c-3p using starBase database. | | | |
| --- | --- | --- | --- |
| **miRNA** | **Predicted lncRNA** | **R-value** | ***p-***value |
| hsa-miR-29C-3P | LINC00511 | -0.368 | 4.62E-36 |
| hsa-miR-29C-3P | LIFR-AS1 | 0.256 | 1.13E-17 |
| hsa-miR-29C-3P | MIR497HG | 0.247 | 1.33E-16 |
| hsa-miR-29C-3P | LINC01224 | 0.123 | 4.95E-05 |
| hsa-miR-29C-3P | HOXA-AS3 | -0.091 | 2.58E-03 |
| hsa-miR-29C-3P | PVT1 | 0.087 | 4.10E-03 |
| hsa-miR-29C-3P | MIAT | 0.048 | 1.13E-01 |
| hsa-miR-29C-3P | MIR193BHG | 0.013 | 6.71E-01 |
